# Electronic Dynamics of a Molecular System Coupled to a Plasmonic Nanoparticle Combining the Polarizable Continuum Model and Many-Body Perturbation Theory

**DOI:** 10.1021/acs.jpcc.2c02209

**Published:** 2022-05-13

**Authors:** Margherita Marsili, Stefano Corni

**Affiliations:** † Dipartimento di Science Chimiche, Università di Padova, via F. Marzolo 1, I-35131, Padova, Italy; ‡ CNR Institute of Nanoscience, Via Campi 213/A, 41125 Modena, Italy

## Abstract

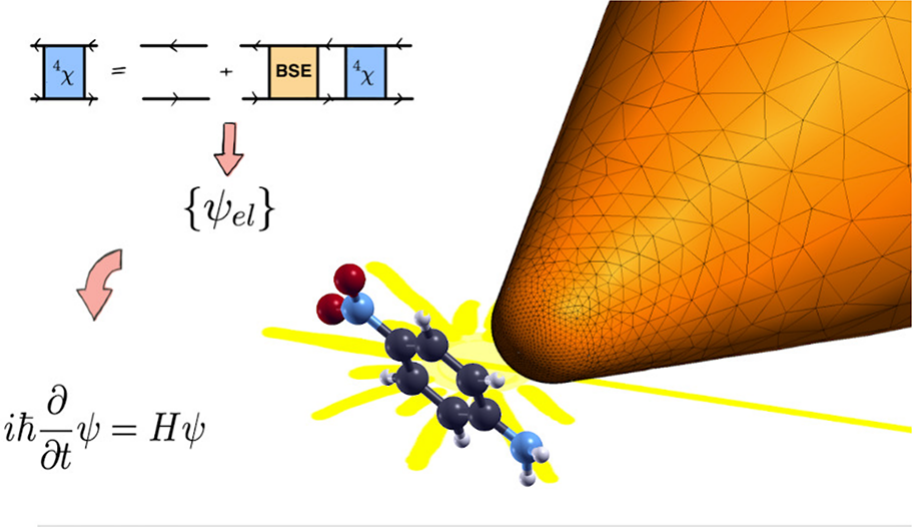

The efficiency of plasmonic metallic nanoparticles in harvesting and concentrating light energy in their proximity triggers a wealth of important and intriguing phenomena. For example, spectroscopies are able to reach single-molecule and intramolecule sensitivities, and important chemical reactions can be effectively photocatalyzed. For the real-time description of the coupled dynamics of a molecule’s electronic system and of a plasmonic nanoparticle, a methodology has been recently proposed (J. Phys. Chem. C. 120, 2016, 28774−2878110.1021/acs.jpcc.6b11084PMC518437028035246) which combines the classical description of the nanoparticle as a polarizable continuum medium with a quantum-mechanical description of the molecule treated at the time-dependent configuration interaction (TDCI) level. In this work, we extend this methodology by describing the molecule using many-body perturbation theory: the molecule’s excitation energies, transition dipoles, and potentials computed at the GW/Bethe–Salpeter equation (BSE) level. This allows us to overcome current limitations of TDCI in terms of achievable accuracy without compromising on the accessible molecular sizes. We illustrate the developed scheme by characterizing the coupled nanoparticle/molecule dynamics of two prototype molecules, LiCN and *p*-nitroaniline.

## 1 Introduction

Surface plasmon resonances (SPRs) are the origins of the tunability and strength of the optical spectra of metallic nanoparticles (NPs) which are thus extremely effective and versatile in harvesting light.^1,2^ One of the most intriguing and exploited properties of SPRs is their ability to concentrate the optical field and amplify it by several orders of magnitude, strongly enhancing light–matter interaction in the proximity of the NP. Such a combination of field enhancement and spatial localization has led to the development of spectroscopies with single-molecule sensitivity such as plasmonic enhanced fluorescence and Raman spectroscopies.^3^ Moreover SPRs can strongly enhance yields and selectivity of several (environmentally and industrially important) chemical reactions, the NP either functioning as an effective photocatalyst or as an antenna that magnifies the activity of a standard catalyst.^4^

The theoretical description of these plasmon-enhanced phenomena is very challenging, and, while the properties of the plasmonic nanoparticle response are already reliably captured using classical approaches where the NP is either treated as a continuum dielectric or as a discrete set of classical atoms,^5,6^ those of the molecule, its response, and its electronic dynamics require full atomistic, quantum-mechanical methods. Thus, multiscale approaches have been applied to describe electronic dynamics of molecules in proximity of plasmonic NPs.^7−12^ In this framework, real-time simulations are especially interesting because they reproduce directly experimental conditions comprising sequences of light pulses and/or pulses with specific shapes, like in ultrafast spectroscopies,^2,13^ and nonlinear optical properties,^14^ and, finally, allow the direct inclusion of relaxation and decoherence effects.^15^

The few existing real-time approaches naturally rely on the finite difference time domain (FDTD) method for the NP polarization coupled to a real-time time-dependent density-functional theory (RT-TDDFT) description of the molecule,^8,12,16^ but recently, also the polarizable continuum model (PCM) method, in its apparent surface charge (ASC) formulation, has been extended to the real-time domain.^11,17^ Originally introduced as a solvation model coupled to quantum-mechanical calculations,^18^ PCM was later adapted to treat NPs (PCM-NP).^7^ Within NP-PCM, the coupling between the classical and quantum parts of the system is of electrostatic nature and is provided by defining an effective Hamiltonian for the quantum system in which an interaction term is added to the vacuum Hamiltonian. The problem to be solved is nonlinear as the added term, induced by the presence of the classical subsystem, self-consistently depends on the charge distribution of the quantum one. Real-time ASC formulation of NP-PCM offers some computational advantages over FDTD approaches^19^ because the electrostatic potential exerted by the quantum system must be evaluated only at the NP surface and not on the entire volume, as FDTD methods require; nonetheless, in view of the overall balance between theoretical accuracy and computational efficiency of the calculation, the choice of the electronic structure method, that treats the quantum part of the system, is also crucial. In particular, real-time PCM-NP has been currently coupled only to TD configuration interaction singles CIS^11,15,20^ or RT-TDDFT,^21^ which are computationally affordable but of limited accuracy in the description of excited state properties of molecules. To be able to gain accuracy without reducing the size of treatable systems, in this work, we extend the real-time formulation of PCM-NP, coupling it with a many-body perturbation theory (MBPT) based description of the quantum subsystem.^22^ MBPT is indeed a valuable framework for the accurate study of excited state properties of extended as well as low-dimensional systems.^23^ Within MBPT, the GW/Bethe–Salpeter equation (BSE) is the standard approach for the description of the electronic structure and optical properties of materials yielding excitation energies in line with high-level theoretical chemistry methods.^23^

Recently, GW/BSE has been successfully coupled to a PCM frequency-domain solvation model.^24,25^ Here, instead, the GW/BSE approach is used to define an effective active-space within which the electronic dynamics of the quantum system develops in real time coupled to the dynamics of the NP.

We first apply this novel scheme to the dipole-switching test molecule LiCN in the presence of a spherical NP, second we study the population and dipole dynamics of the prototypical push–pull *p*-nitroaniline (PNA) molecule in proximity of a tip-shaped silver nanoparticle.

The paper is organized as follows: first the PCM and GW-BSE approaches are briefly reviewed to recall the main equations that are involved in our computational scheme and the physical framework that they define. Second, the formalism for the coupled NP-molecule dynamics is introduced; finally, in Results and Discussion, we apply the method to the cases of LiCN and PNA molecules.

## 2 Methods

### 2.1 General Formalism for the Coupled Nanoparticle/Molecule System

The typical size of NPs involved in nanoplasmonic devices ranges from tens to several hundreds of nanometers. The full quantum treatment of objects of such a size is not affordable, and, eventually, it is not needed because a classical treatment already yields a good description of the NP optical response even at the nanometric scale.^26^ Indeed, even if quantum effects such as those related to the electron confinement and the nonlocal nature of the electronic screening are neglected in classical electromagnetic simulations, they still provide a very adequate framework to address local field distribution even in extreme cases like the very proximity of atomic-scale features of plasmonic NPs,^26−28^ such that even submolecularly resolved photoluminescence maps could be accurately predicted to an almost quantitative degree.^29^ The method employed in the present work for the description of the NP is an extension of the PCM in its integral equation formulation (IEF)^19^ originally developed for the description of a (polarizable) solvent and later generalized to the description of metallic NPs (PCM-NP).^7^ Within the PCM-NP approach, the NP is described as a continuous medium characterized by a frequency-dependent dielectric function and a (complex) shape. When a molecule is set in proximity of the NP and the system is lit up, both the incident radiation and the changes in the electronic density of the quantum system polarize the NP. In turn, the polarized NP creates time-dependent electric fields that act on the molecule together with the incoming radiation. The two processes happen together and must be solved self-consistently. This methodology is described in its details in ref (18); here, we simply review its main physical contents and the equations needed to introduce the present work.

Within PCM-NP, derived in the quasistatic limit, the response of the NP is entirely expressed in terms of potentials generated by a fictitious (or apparent) charge distribution laying on the NP surface.

To be able to treat arbitrary NP shapes, the surface is described using a discrete mesh. Each element of this mesh, called tessera, is characterized by a representative point *s⃗*_*i*_, the element area *a*_*i*_ and a charge *q*_*i*_. The Hamiltonian of the quantum system in proximity of the NP can then be expressed as
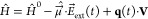
1where *H*^0^ is the isolated quantum system, μ⃗̂ is the dipole operator, and *E⃗*_ext_ is the incoming electric field. Bold quantities are vectors or matrices in the tesserae space: each element of *q*_*i*_ is the apparent charge of the *i*th tessera, and each element of *V*_*i*_ is the electrostatic potential of the quantum system (generated by its electrons and nuclei) at the *i*th tessera position.

At each time *t* the apparent charges **q**(*t*) are given in terms of a response function **Q**, a matrix in the tesserae space, which is nonlocal in time due to the frequency dependence of the dielectric function, namely:
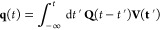
2The kernel **Q** of this integral equation is fully determined by the NP features, namely, its dielectric function, and its geometry, and can, in principle, be obtained by Fourier transforming its frequency-domain counterpart, although we shall use a different strategy.

From eq 2, it is clear that the value of the apparent charges at a given time *t* depends on the full history of the system. When the NP dielectric function takes a Drude–Lorentz form,^30^ namely:
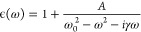
3an equation of motion for the apparent charges depending only on the properties of the system at the instant *t* can be obtained.^11^
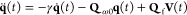
4where the form of the matrices **Q**_ω0_ and **Q**_f_ depends on the Drude–Lorentz parameters and on the geometry of the NP as shown in ref.^11^.

It is worth noting that besides the Drude–Lorentz form, which will be used in the present work, a recent formulation, employing a generic (typically the experimental one) ϵ(ω), can be used and still allows an equation of motion formulation of the ASC dynamics.^17^ Thanks to eq 4, the charges evolution in time **q**(*t*) can be numerically obtained.

### 2.2 GW-BSE Approach

Excited states of the molecule will be addressed using the GW-BSE method, derived within MBPT.^22^ The GW/BSE method, traditionally used by the community of inorganic solid state physics to introduce the effect of electronic correlation in the calculation of electronic and optical properties of materials, is rapidly gaining importance also among chemists yielding accurate oscillator strengths, 0–0 energies of quality comparable to ADC(2) and CC2, and correctly describing, at the same time, charge transfer and local excitations in molecules.^23,31^

Within the standard approach, GW-BSE calculations proceed in three logical and computational steps: (1) the determination of the electronic ground state, typically within density-functional theory;^32^ (2) the determination of single-particle excitation energies by computing GW self-energy corrections to the DFT-KS single-particle energy levels;^22^ (3) the determination of neutral excitation energies (i.e., excitations with unchanged number of electrons in the systems, such as optical excitations) and of the optical properties by the solution of the BSE.^22^ In the following, we briefly recall this last step, its main physical insights, and the equations that are needed to be able to follow the present work; comprehensive reviews on the GW-BSE method can be found in refs (22, 23, and 33).

As already mentioned, the BSE method is built on top of an accurate description of electronic charged excitations, i.e., excitations in which an electron is added or removed from the system, such as in direct and inverse photoemission experiments. These excitations are described as quasiparticles (QPs), “dressed” electrons or holes, characterized by their respective energies (and line-widths) and amplitudes. Typically, starting from the DFT-KS scheme, the energies are obtained as first-order corrections to the KS energies, while the amplitudes are kept equal to the single-particle KS wave functions.

When light is shone on the system, it promotes such quasiparticles from occupied to empty levels. If the interaction between the quasielectron and quasiholes was neglected, neutral excitations accessible by light would simply be single quasiparticle transitions at energies given by the corresponding quasiparticle energy differences.

For systems where the electronic screening is not very effective, for example, in molecules and low-dimensional systems, this approximation fails badly,^22^ and the interaction within the excited electron–hole (e–h) pairs cannot be neglected.

In the GW-BSE approach such e–h interaction is approximated by a bare repulsive contribution term, that takes its origin from the classical Hartree potential *K*^*x*^, and an attractive, static, screened Coulomb interaction term *K*^*d*^, originated from the exchange and correlation contribution to the QP interaction.^33,34^

As a result of the e–h interaction, the optical oscillator strengths are obtained from specific combinations of single quasiparticle transitions located at specific energies. In the standard implementations of the GW-BSE method, such combinations and energies are obtained by diagonalizing an effective two-particle Hamiltonian in the e–h transition space,^33^ namely:
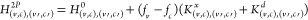
5where *v* and *c* stand for full sets of quantum numbers that identify the single QP states and *f*_*v*_ and *f*_*c*_ are the corresponding occupations. In eq 5*H*^0^ describes the noninteracting e–h couple:
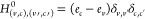
6*K*^*x*^ is given by
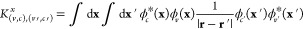
7*K*^*d*^ is the direct, screened attractive term:
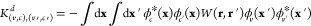
8*e*_*i*_ are the QP energies, **x** stands for spin and spatial coordinates, ϕ_*i*_ are single-particle wave functions, and *W*(**r**,**r**′) is the screened Coulomb interaction given by
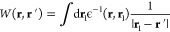
9where ϵ^–1^(**r**, **r**_1_) is the microscopic dielectric function of the system. In the general case, *c* and *v* would identify all the possible single QP particle states, but in the current implementation we have used the Tamm–Dancoff approximation which decouples excitations and de-excitations, and in this case *v* and *c* identify occupied and empty states, respectively.^22^ The diagonalization of eq 5 provides a set of eigenvalues *E*_λ_ with the corresponding eigenvectors *A*_*cv*_^λ^, that allow us to define the BSE electron–hole eigenstates:
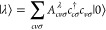
10While the BSE eigenvalues correspond to the optical excitation energies of the system, the electron–hole eigenstates are used to obtain the corresponding oscillator strengths, as shown for example in details in refs (31 and 33) and thus to build up the optical absorption spectrum. In this work, we employ the BSE eigenstates as approximate excited states of the system so that, referring to eq 1:
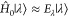
11with energy referring to the ground-state Slater determinant |λ = 0⟩ = |0⟩.

### 2.3 Static Equilibration of the Coupled Nanoparticle/Molecule System

Once the molecule and the NP are placed close one to the other, even in the absence of an external field, the mutual interaction alters their conditions from the isolated case. In particular, the NP develops a polarization responding to the ground-state charge distribution of the molecule, and this polarization, in turn, modifies the ground and excited states of the molecule and its energy spectrum. These changes are self-consistently computed as a preliminary step for the dynamics.

More specifically, the equilibration procedure follows these steps:

- Starting from a GW-BSE calculation in vacuum, a finite set of |λ⟩_vac_ states is chosen as the molecule “active space”.
- Within the chosen active space, the total energy of the NP/molecule system is self-consistently minimized starting from the molecule in its vacuum ground-state |0⟩_vac_ in proximity to the nonpolarized NP. As a result, the NP is provided of a set of starting polarization charges **q**_eq_^GS^, while the molecule is in a novel ground-state |0⟩_eq_ = ∑_λ_*a*_λ_^GS^|λ⟩_vac_.
- The NP polarization, frozen at the ground-state value **q**_eq_^GS^, provides a modified molecular Hamiltonian *Ĥ* = *Ĥ*^0^ + **q**_eq_^GS^·**V** that is diagonalized in the molecular active space, obtaining a new set of excited states |λ⟩_eq_ = ∑_λ′_*a*_λ′_^λ^|λ′⟩_vac_ and excitation energies *E*_λ_^eq^. These correspond to the frozen states used in ref (11).

The novel molecular ground-state |0⟩_eq_ and NP polarization charges **q**_eq_^GS^ represent the starting point of the coupled dynamics which develops within the active space spanned by the set {|λ⟩_eq_}. In the following, we will refer to the sets {|λ⟩_eq_} and {*E*_λ_^eq^} simply as {|λ⟩} and {*E*_λ_} taking the equilibration procedure for granted.

### 2.4 Coupled Nanoparticle/Molecule Dynamics

The coupled nanoparticle/molecule dynamics is obtained by simultaneously propagating in time the equation of motion for the apparent charges (eq 4) and the time-dependent Schrödinger equation governed by the Hamiltonian in eq 1 for the molecule. At any time *t*, the state of the molecule is expanded in terms of the set {|λ⟩}:
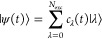
12where *N*_exc_ is the number of excited states taken into account in the dynamics. The time-dependent Schrödinger equation then becomes a coupled set of equations for the coefficients *c*_λ_(*t*):
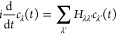
13where *H*_*λλ*′_ are the molecule’s Hamiltonian matrix elements:
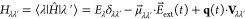
14μ⃗_*λλ*′_ and **V**_*λλ*′_ are the transition dipole moment and transition potential on the tesserae, respectively.

For the chosen form of {|λ⟩} (see eq 10), the transition matrix element of any single-particle operator *Ô* = ∑_*i* = 1_^*N*^*ô*_*i*_ (*N* being the total number of electrons) is given by
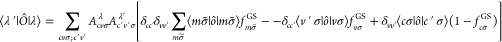
15The transition dipoles are obtained using *ô*_*i*_ = −*r*_*i*_, while the −*Tth* component of the **V**_*λλ*′_ vector in the tesserae space of eq 14 is given by
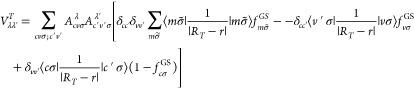
16

## 3 Results and Discussion

### 3.1 Computational Details

DFT and GW-BSE calculations were performed as implemented in the MOLGW.^35^ Real-time dynamics, under the influence of an external pulse, were simulated using the WaveT/TDplas suite either in vacuum or coupled to a plasmonic NP.^36^ For the LiCN molecule, the 6-31G basis set^37^ and the cam-b3lyp^38^ DFT exchange-correlation (xc) functional were used. Single-shot G_0_W_0_ calculations, for the single-particle energy levels, and BSE calculations, for the determination of the neutral excited states, were carried out employing the whole transition space allowed within the chosen basis set. Transition dipoles, μ⃗_*λλ*′_, and potentials, **V**_*λλ*′_, between the ground state and excited states and within excited states were computed for excitation energies up to 8.6 eV (15 excitations in total) and used for the dynamics.

For the PNA molecule, the cc-pVTZ basis set^37^ and the PBE0^39^ DFT xc functional were used. The geometry was obtained from the full optimization, at the DFT-PBE0, level of the chemical structure present in the NIST Web site.^40^ Self-consistent G_*n*_W_*n*_ calculations, for the single-particle energy levels, and BSE calculations, for the determination of the neutral excited states, were carried out employing the whole transition space allowed within the chosen basis set. Transition dipoles, μ⃗_*λλ*′_, and potentials, **V**_*λλ*′_, between ground state and excited states and within excited states were computed for excitation energies up to 10 eV (28 excitations in total) and used for the dynamics.

### 3.2 LiCN

The presence of a bright, charge-transfer, doubly degenerate excitation in its optical spectrum makes LiCN an ideal test system for the study of optical dipole switching.^41−43^

#### 3.2.1 Static Effects of the NP on LiCN

**Figure 1:**
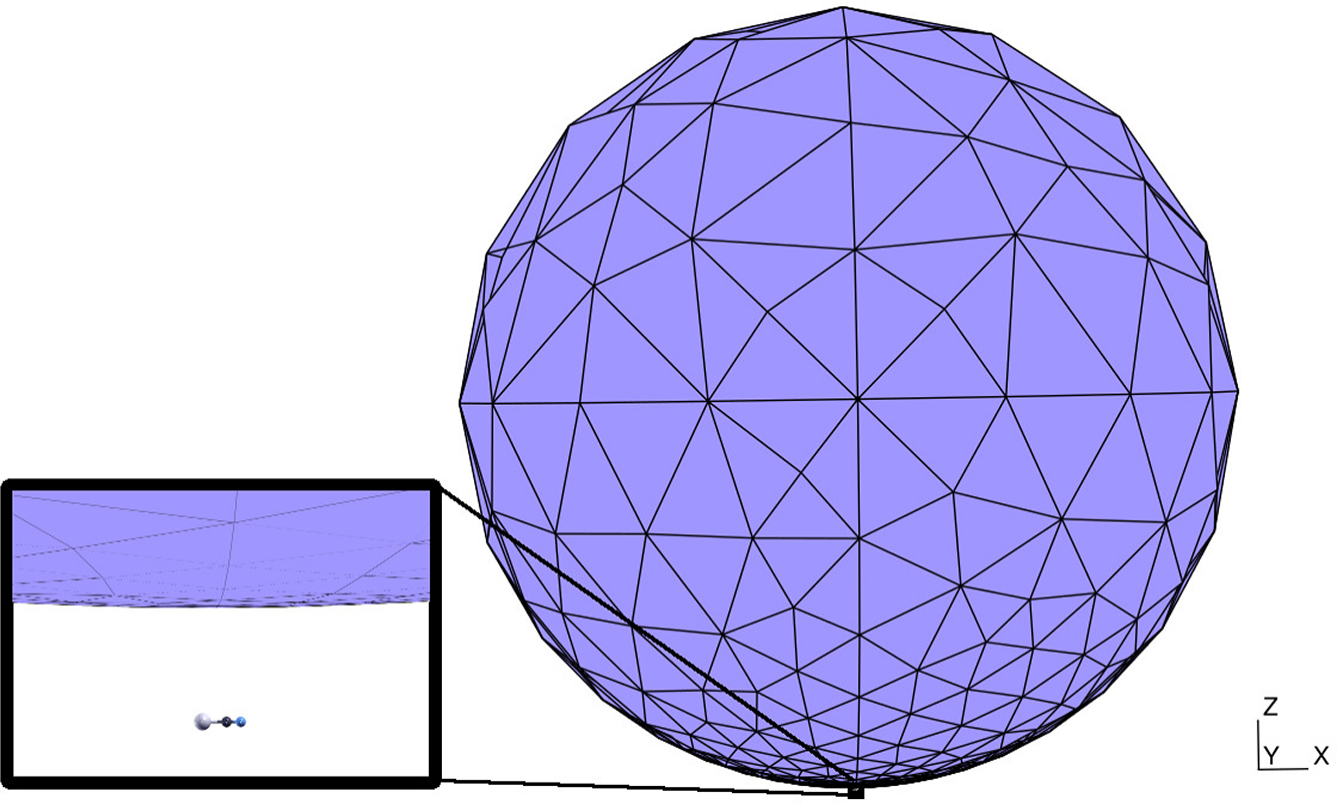
LiCN/NP geometry: the molecule is placed at a representative distance from the spherical NP. The gray, black, and blue balls represent Li, C, and N atoms, respectively.

We place the LiCN molecule in proximity to a spherical NP of 5 nm diameter, as shown in Figure 1. The axis of the molecule is parallel to the plane tangent to the NP at its closest point to the molecule. The NP–molecule distance is varied between 3 and 100 Å. The dielectric function of the NP is given by eq 3, with *A* = 0.124 a.u., ω_0_ = 0 a.u., and γ = 0.0075 a.u. These parameters were chosen in order to match the NP resonance with the energy of the dipole-switch transition. As can be seen in Figure 1, the NP tessellation, obtained using the gmsh software,^44^ was built not to be uniform since the mesh element size (mes) decreases smoothly from 2.1 nm at the furthest point from the molecule to 0.1 nm at the closest point. The convergence with respect to the mesh parameters has been checked as shown in the Supporting Information.

**Figure 2:**
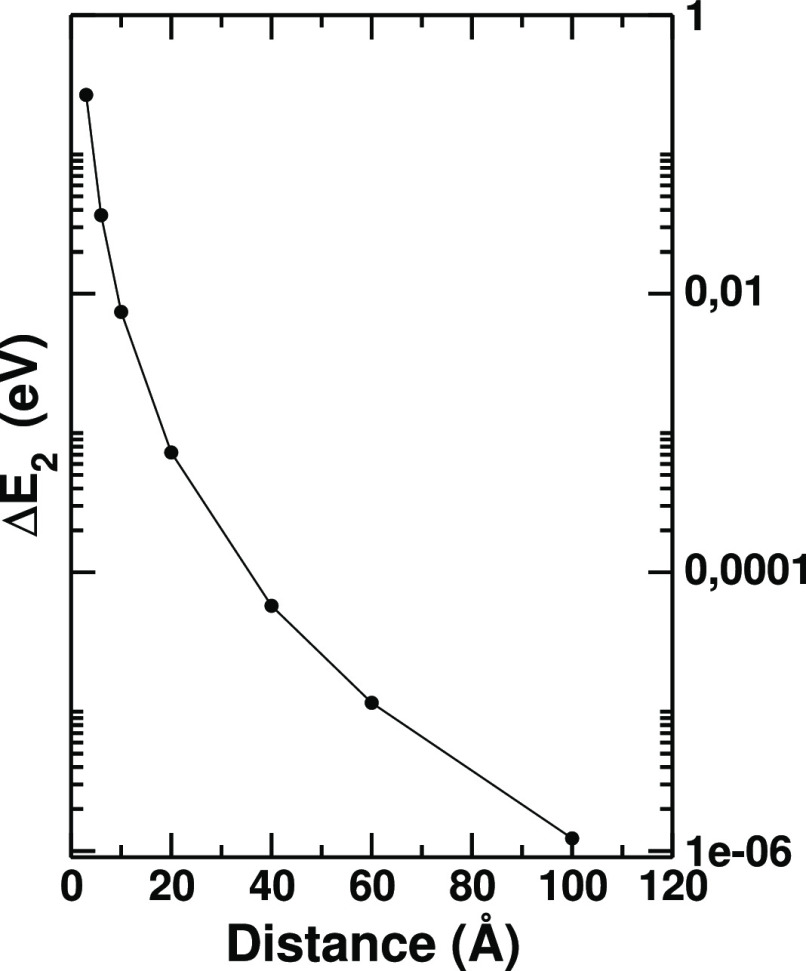
*ΔE*_2_ = *E*_2_^NP^ – *E*_2_^vac^, i.e., the variation of the energy of the dipole-switch excited state upon equilibration with the NP, varying the NP/molecule distance.

In Table 1, the dipole moments induced in the NP by the molecule after the equilibration procedure described in the theory section for different NP–molecule distances are shown. As the molecule is moved further away from the NP, the induced dipole moment decreases as expected. In the same table, we report the analytic image dipole moment induced in a 5 nm spherical metallic NP by a point-charge dipole of the same magnitude of that of the ground-state LiCN molecule oriented and set at the same distance with respect to the NP. The analytic results show a good agreement with the computed values confirming the quality of the NP tessellation. The effect of the NP on the energy spectrum of the molecule is shown in Figure 2, where *ΔE*_2_ = *E*_2_^NP^ – *E*_2_^vac^, i.e., the variation of the dipole-switch transition energy with respect to the isolated case, is reported. We recall that *E*_λ_^NP^ values are obtained diagonalizing, within the chosen active space, a molecule Hamiltonian where the interaction with the NP is present, the NP polarization being frozen at its ground-state self-consistent value. The variation *ΔE*_2_ is positive at all distances, reflecting the fact that the frozen polarization of the NP stabilizes the ground state of the molecule more than the excited state. Significantly, although the energy difference decays extremely rapidly with the NP–molecule distance, for the 3 Å case it can be as large as ∼0.3 eV, which is consistent with a ∼0.2 eV interaction energy of the classical point-charge dipoles of the same magnitude set at the same distance as those of the equilibrated ground-state molecule and NP.

**Table 1:** Dipole Moment μ_NP_ Induced by the Molecule within the NP after the Equilibration Procedure Compared with the Analytic Image Dipole for Point-Charges Dipole Set at the Same Distance

dist (Å)	3	6	10	20	40	60	100
μ_NP,PCM_ (a.u.)	2.95	2.49	2.01	1.26	0.59	0.32	0.12
μ_NP,Image_ (a.u.)	2.96	2.51	2.04	1.29	0.61	0.33	0.13

#### 3.2.2 Coupled NP/Molecule Dynamics

**Figure 3:**
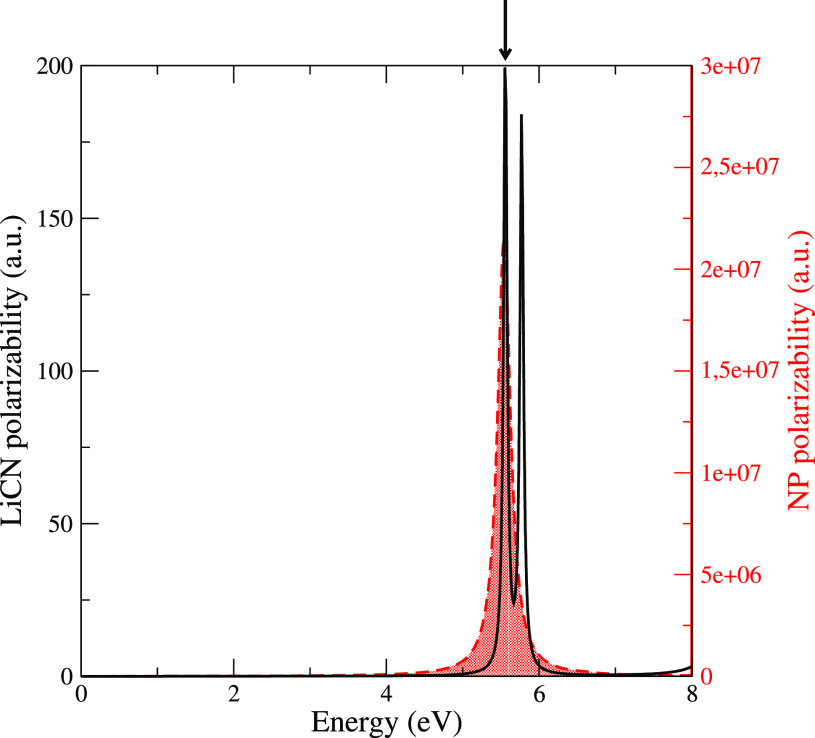
Dynamical polarizability (along the *z* direction) of the LiCN molecule (black full line, left axis) and of the NP (red dashed line, right axis). The arrows points the energy of the LiCN dipole-switch transition.

**Figure 4:**
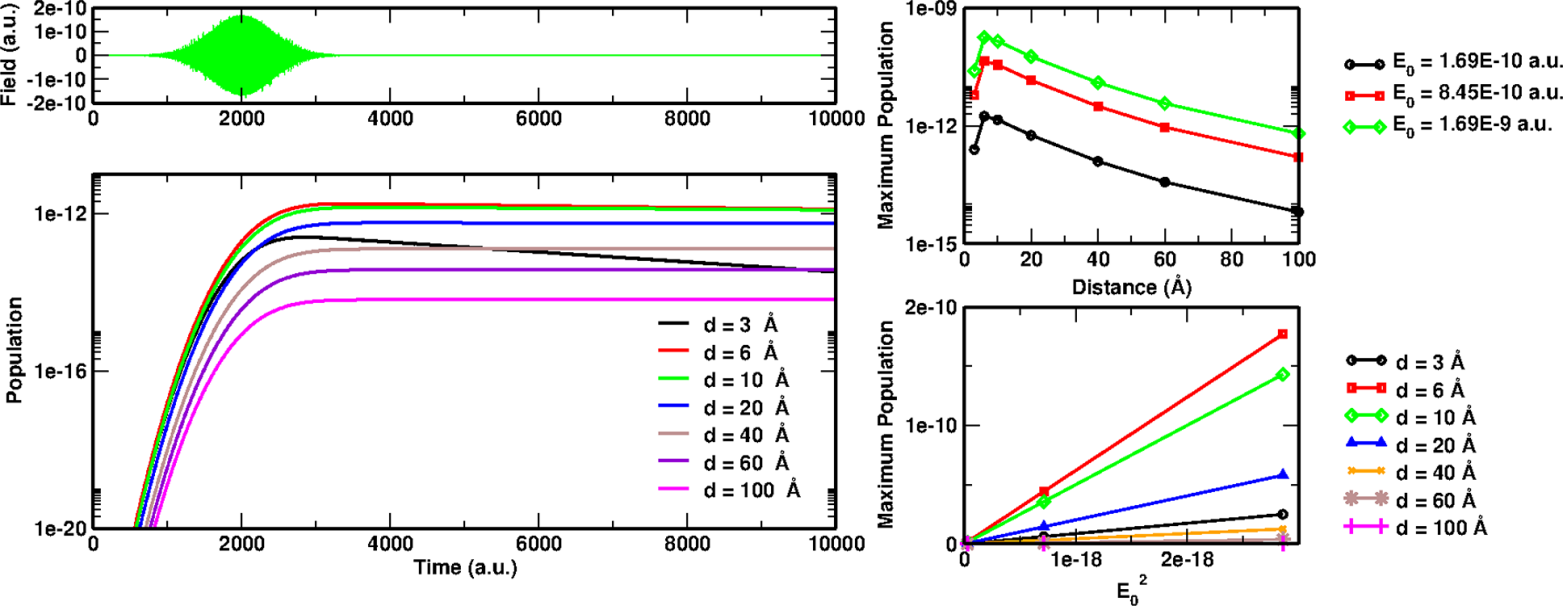
Left: top panel - external pulse; bottom panel - dynamics of the dipole-switch states population for *E*_0_ = 1.69 × 10^–10^ a.u.. Right: top panel - maximum population vs the NP/molecule distance for the three field intensities; bottom panel - maximum population vs field intensity.

The NP optical spectrum is shown in Figure 3. The dielectric constant parameters were tuned in order for the NP resonance to match the LiCN dipole transition energy in vacuum, shown in the same figure. We propagate eq 4 and eq 13 for the LiCN molecule in proximity of the spherical NP in the presence of a Gaussian enveloped pulse in resonance with the dipole-switch transition energies provided by the initial equilibration procedure:
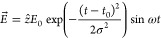
17The pulse is polarized along the *z* direction, perpendicularly to the molecule axis, σ = 10 fs and *t*_0_ = 50 fs. We employed three different fields amplitudes *E*_0_ = 1.69 × 10^–10^ a.u., *E*_0_ = 8.45 × 10^–10^ a.u., and *E*_0_ = 1.69 × 10^–9^ a.u. (corresponding to 10^–3^ W/cm^2^, 2.5 × 10^–2^ W/cm^2^ 10^–1^ W/cm^2^ intensities respectively). As shown in the Supporting Information, at these field intensities, the inclusion of the first four energy levels is enough to get converged electronic dynamics. The dynamics of the population of the dipole-switch states in resonance with the pulse is shown in Figure 4 for the *E*_0_ = 1.69 × 10^–10^ a.u. case.

For all the NP/molecule distances, the population of the dipole-switch states increases during the pulse reaching a maximum value which stays approximately constant after the pulse except for the 3 Å case where the excited states population steadily decreases. Indeed, the electronic dynamics does not include any dissipative channel that would allow the excitation to relax, but at a 3 Å distance (and also in the 6 Å case, but with longer time-scales) the coupling between the NP and the molecule is very strong, and the molecule is able to quickly relax through the NP dissipative dynamics. As shown in the Supporting Information, this is also manifest in the high fields case, where, at a 3 Å NP/molecule distance, the Rabi physics is that of two-level system strongly coupled to other degrees of freedom. In the top left panel of Figure 4, the maxima of the population are displayed against the NP/molecule distance for the three field intensities. In all of the cases, the maximum value does not follow a monotonic trend with the NP–molecule distance. The reason for this behavior is that the field amplification is not monotonic as well. This is mainly caused by the fact that the pulse energy is set at slightly different values for each NP–molecule distance in order to match the equlibrated molecule excitation energy, thus sampling at slightly different values the the NP response. This issue is also discussed the analysis of the high field regime and Rabi oscillations present in the Supporting Information. At these field intensities, the response of the system is linear as shown in the bottom left panel of Figure 4.

### 3.3 PNA

**Figure 5:**
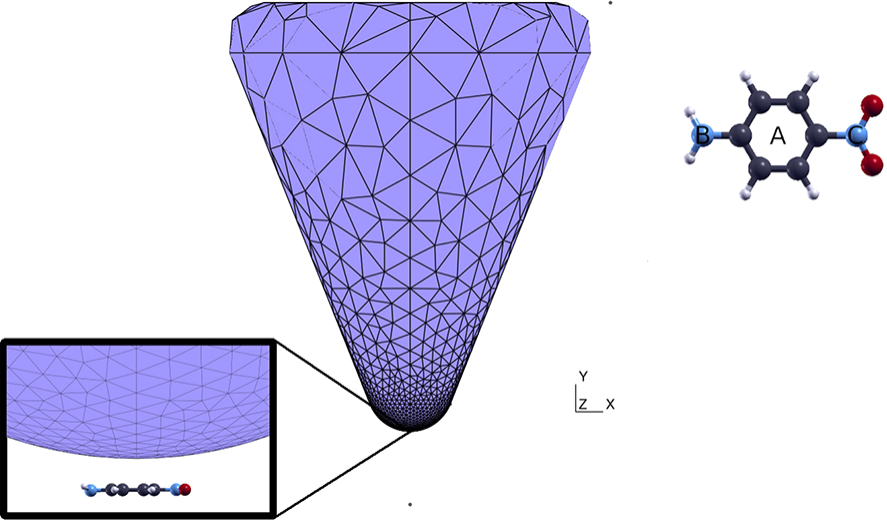
PNA molecule (top view) with labels referring to the NP tip; tip NP and PNA molecule geometry (side view); in the inset the zoom of the tip vertex is provided. Blue, red, black, and light gray balls correspond to N, O, C, and H atoms respectively.

*p*-Nitroaniline (PNA), shown in Figure 5, is a prototypical planar push–pull molecule, a model for more complex dyes, characterized by a benzene ring with an electron-donating amine group NH_2_ linked to an opposite end with respect to an acceptor nitro group NO_2_.

We study the excited state population dynamics in realistic conditions. In particular, the molecule is set in the presence of a tip-shaped nanoparticle under the influence of a Gaussian enveloped, 10 fs pulse in resonance with PNA first bright excitation described by eq 17. The pulse is polarized perpendicularly to the molecule plane (*xz*), *E*_0_ = 1.69 × 10^–9^ a.u. (corresponding to a 10^3^ W/m^2^ intensity), σ = 10 fs, and *t*_0_ = 50 fs. The nanoparticle, modeled as a tip, shown in Figure 5, is a silver cone with rounded edges: the cone height is 5 nm, while its basis radius measures 2 nm. The cone axis is oriented along the *y* direction, thus perpendicularly to the PNA molecular plane, at a 3 Å distance from it. A Drude dielectric function has been used with silver parameters taken from ref (11). The nanoparticle surface was discretized with a mesh made of 2428 tesserae whose dimensions decreased when approaching the cone tip. As a possible virtual experiment, we scanned the molecule by positioning the tip over three different spots, namely, in the middle of the benzene ring (position A), on top of the N atom belonging to the amine group (position B), and on top of the N atom belonging to the nitro group (position C). In Figure 5, the position of the tip for each geometry is shown.

**Figure 6:**
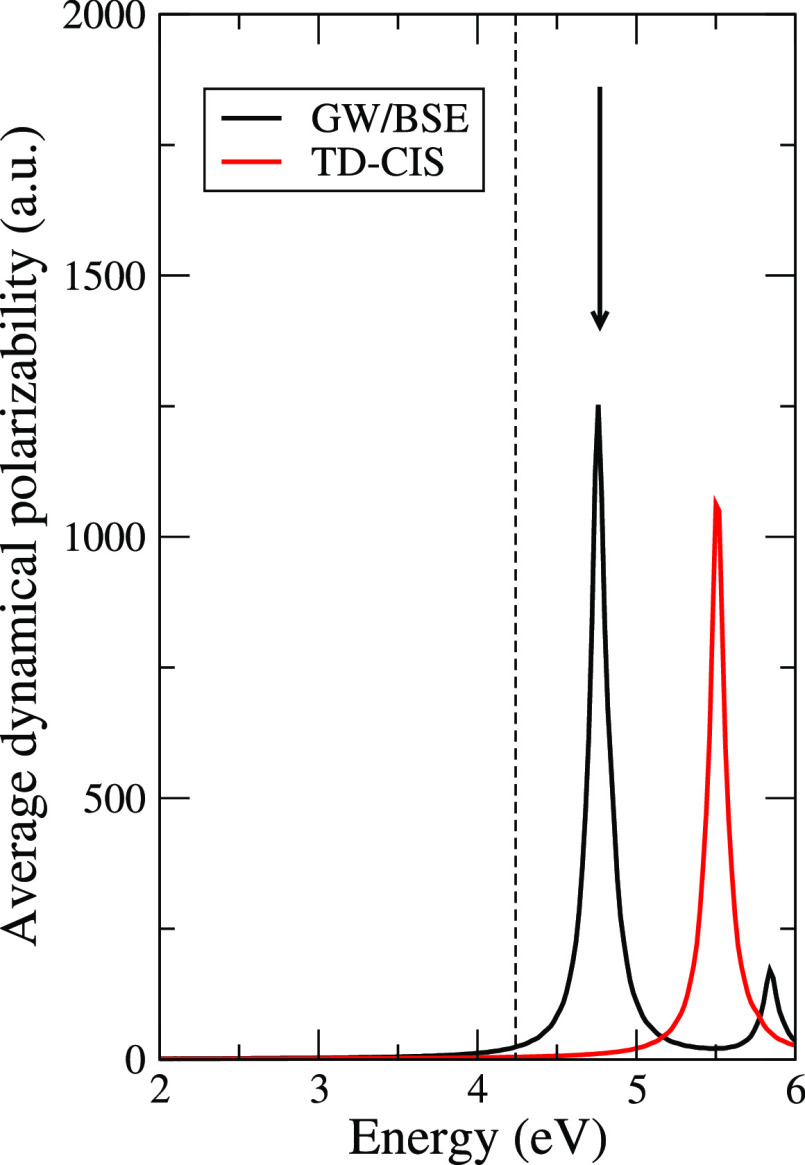
PNA dynamical polarizability, averaged along the three polarization directions. Black: GW/BSE calculation; red TDCIS calculation. Black arrow: external pulse frequency; dashed line first bright excitation reported in visible-UV experimental absorption spectra.^45^

Shown in Figure 6 are the optical absorption spectrum and the chosen external electric field is in resonance with the first bright peak around 4.77 eV. For the sake of comparison, in the same figure, the TDCIS spectrum is also presented: the first bright peak is in this case found at 5.5 eV, while experimental visible-UV absorption spectra^45^ report a first excitation with a finite oscillator strength at 4.24 eV (shown as a dashed line in the figure) in better agreement with the GW/BSE excitation energy. It is worth noting that for the purpose of this work the accuracy of the GW/BSE calculation was not pushed as far as possible: we employed the Tamm–Dancoff approximation (which is known to have large impact on finite systems^46^), and we performed only an eigenvalue G_*n*_W_*n*_ self-consistency without updating the quasiparticle wave functions.^23^

**Figure 7:**
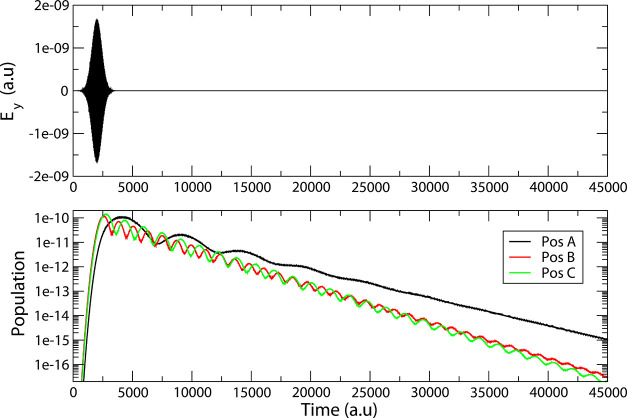
Top panel: *y*-component of the incident field. Bottom panel: time evolution of the resonant state population: black: tip at position A; red: tip at position B; green: tip at position C.

In the bottom panel of Figure 7, the population of the excited state in resonance with the external field is shown for the three geometries. At a 3 Å distance, the population dynamics is sensitive to the tip position: position A is characterized by a slower dynamics in which the excited state population takes longer time to rise and decay than in positions B and C; faster oscillations are superimposed to the general rising/decaying trend in the B and C cases with respect to A. Indeed, the maximum population is reached with a delay of ∼2000 au (∼48 fs) with respect to the field maximum for position A, whereas such delay is ∼580 au (∼14 fs) and ∼760 au (∼14 fs) for the B and C positions respectively. By fitting the exponential decay part of the population dynamics in the range between 25 000 au and 50 000 au, we obtain decay rates of 2.7 × 10^–4^ a.u. for position A, 3.2 × 10^–4^ a.u. for position B, and 3.5 × 10^–4^ a.u. for position C, corresponding to 90, 75, and 70 fs lifetime, respectively. Such sensitivity with respect to the tip position suggests that in experiment where the relative NP/molecule position is not fully controlled an average lifetime is what is possibly accessed. Changes in the molecular excited state lifetimes as a function of the tip position were recently shown experimentally.^29,47^

## 4 Conclusions

In conclusion, to describe the electronic dynamics of a molecule in proximity of a plasmonic NP, we coupled a real-time PCM approach for the NP to a MBPT-based description of the excited states of the molecule. The molecule’s excitation energies are obtained within the GW-BSE approach, and the excited states are approximated by a linear combination of singly excited Slater determinants whose coefficients are given by the eigenvectors of the effective two-particle BSE Hamiltonian from which transition dipoles and potentials on the tesserae are computed. The coupled equation of motion for the NP apparent surface charges and for the molecule electronic system are then propagated in time. By using MBPT for the description of the electronic system, the proposed development allows an accurate treatment of charge-transfer excitations and energy level alignments at interfaces^23,48,49^ going beyond the standard implementations of TDDFT and a better description of optical properties with respect to TDCIS, which is what was coupled to the real-time formulation of NP-PCM up to now. At the same time, a favorable overall scaling with the system size is maintained.^23,50^ We applied this methodology to two prototype systems, namely, the dipole-switch LiCN molecule in the presence of a spherical NP and the push–pull PNA coupled to a tip-shaped silver NP. While in the present work, the NP dielectric function takes a Drude–Lorentz form, the formulation for using a generic ϵ(ω) is already available and implemented.^17^

The LiCN molecule was positioned at increasing distances with respect to the NP, and the external field was set in resonance with the energy of the doubly degenerate dipole-switch transitions. A tip-shaped NP probed the response of the PNA molecule at three different positions: at the center of the benzene ring, on the amine group, and on the nitro group. We observe a strong sensitivity of the response in terms of resonant state population dynamics as a function of the tip position.

## Supporting Information Available

LiCN: geometry and GW-BSE optical properties, NP mesh convergence, high field regime and Rabi oscillations, convergence on active-space size. PNA: GW-BSE electronic structure and optical properties, NP and active-space-size convergence (PDF)
