# Supplementary material for: Electronic Dynamics of a Molecular System Coupled to a Plasmonic Nanoparticle Combining the Polarizable Continuum Model and Many-Body Perturbation Theory
Source: J Phys Chem C Nanomater Interfaces. 2022 May 13;126(20):8768–76. doi: 10.1021/acs.jpcc.2c02209 (PMC9150096; doi:10.1021/acs.jpcc.2c02209)
Supplement: Supplementary file 1 — jp2c02209_si_001.pdf [file jp2c02209_si_001.pdf]

Supporting Information: Electronic Dynamics of a Molecular System Coupled to a Plasmonic Nanoparticle Combining the Polarizable Continuum Model and Many-Body Perturbation Theory

Margherita Marsili[*,†] and Stefano Corni[†,‡]

†*Dipartimento di Science Chimiche, Università di Padova, via F. Marzolo 1, I-35131, Padova, Italy*

‡*CNR Institute of Nanoscience, Via Campi 213/A, 41125 Modena, Italy*

E-mail: margherita.marsili@unipd.it

1 LiCN geometry and GW-BSE optical properties

LiCN molecular axis is oriented along the x direction with the x-axis going from the Li to the N atom. The C-N bond length is equal to 2.168 a.u., while C-Li bond length to 3.683. At the DFT cam-b3lyp level the electronic gap of the molecule is 7.8 eV, which becomes 9.1 eV taking into account G_0W_0 corrections. As found in previous calculations[1] both HOMO and LUMO are non degenerate states at the DFT level, at odds with HF methods that predict a doubly degenerate HOMO,[1] and G_0W_0 do not change the DFT level ordering. The optical gap shrinks is 4.9 eV, 4.2 eV smaller than the electronic one, due to the strong electron-hole interactions, related to the low dimensionality of the system. The ground state dipole of the system, is -3.53 a.u. in agreement with previous calculations.[1,2] The BSE excitation

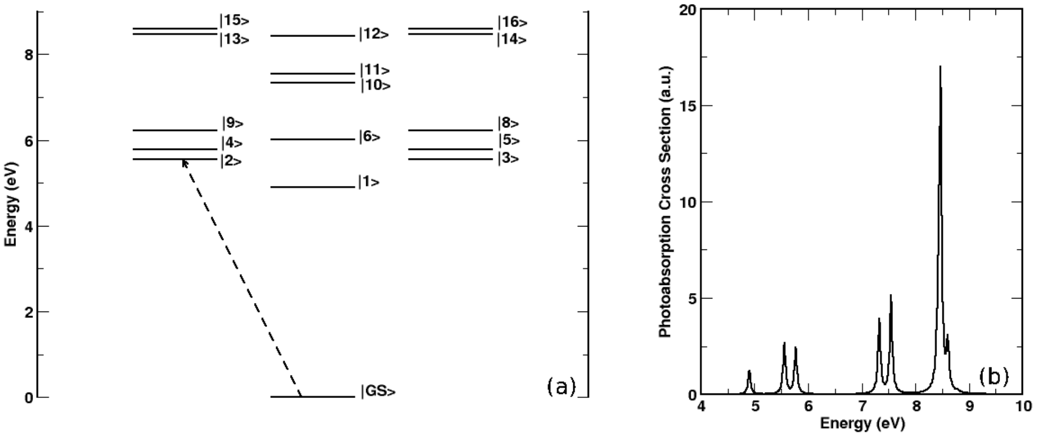

Figure S1: (a) BSE energy levels. The dashed arrow shows the transition in resonance with the applied external field which is mostly excited by it, due to the light polarization along the z axis. (b) GW-BSE photoabsorption cross section.

levels included in the dynamics are presented in Fig. 1, panel (a). The energy levels show a qualitative good agreement with previous configuration-interaction singles (doubles) (TD-CIS(D)) calculations.[2] In panel (b) of Fig. 1 the GW-BSE photoabsorption cross section is shown. Tab. 1 reports the energy, dipole moment x-component μ_x and the y and z components of the transition dipole moments of state 2 and 3, in resonance with the applied

field. Clearly a z-polarized radiation will populate mainly state 2.

Table S1: Energy, dipole moment x-component μ_x, transition dipole moments y and z components $\mu_y/z^{|0\rangle\rightarrow|I\rangle}$ of the doubly degenerate excited states in resonance with the external field.

State	E (eV)	μ_x (a.u.)	$\mu_y^{|GS\rangle\rightarrow|I\rangle}$ (a.u)	$\mu_z^{|GS\rangle\ \rightarrow|I\rangle}(a.u.)$
$|2\rangle$	5.6	2.24	0.078	0.451
$|3\rangle$	5.6	2.24	0.451	0.078

2 LiCN NP mesh convergence

For the determination of the optimal mesh the LiCN molecule is aligned along the x axis, and the center of the NP is located along the z axis as shown in Fig. 2. The presence of the NP breaks the rotational symmetry along the x-axis.

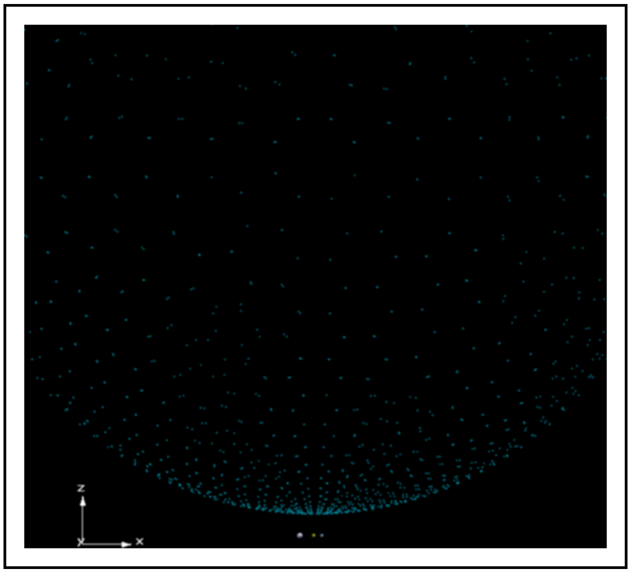

Figure S2: LiCN/NP geometry adopted for the determination of the optimal mesh.

The convergence of the NP tessellation was tested at 3 Ådistance, where the mutual NP-molecule interaction is most sensitive to the details of the tessellation close to the molecule. Five tessellations were compared: a uniform mesh with mesh element size (mes) fixed to 1.2 nm, and four non uniform meshes with decreasing mes upon reaching the surface point

closest to the molecule. In these cases the mesh was determined by fixing the mes couple t-b where t stands for the mes of the furthest top point of the NP and b for the closest bottom point.

As already mentioned in the text, also without any external field, the presence of a metallic NP in proximity of the molecule affects the molecule's spectrum and ground state polarization; in turn the NP acquires a finite polarization. For the study of the optimal tessellation we included only the ground and the first three excited states. In Tab. the modified energies of the second and third excited state E_2 and E_3, as well as degree of degeneracy breaking$\Delta_E = E_3 - E2$, the sum of the square modulus of the z-component of the transition dipole moments of the two states $d_z^2 = |\langle 0|\hat{d}_z|2\rangle > |^2 + \langle 0|\hat{d}_z|3\rangle > |^2$, and the NP induced dipole moment $\vec{\mu}$ are reported.

Table S2: Variation with respect to the NP surface tessellation of the self-consistent excitation energies of the second and third excited state, E_2 and E_3 respectively, of the symmetry breaking due to the tessellation $\Delta_E = E_3 - E2$, of the sum of the square modulus of the z-component of the transition dipole moments of the two states d_z^2, and of the statically induced NP dipole moment μ_i (in a.u.). The different mesh are characterized by the mes couple t-b (in nm), the total number of mesh elements is provided in parenthesis.

Mesh	1.2-1.2 (640)	1.6-0.3 (666)	2.1-0.1 (624)	1.6-0.1 (970)	1.1-0.1 (1712)
E_2 (eV)	5.656	5.814	5.826	5.827	5.828
E_3 (eV)	5.657	5.816	5.828	5.829	5.829
Δ_E (meV)	0.46	1.62	1.44	1.38	1.45
d_z^2 (a.u)	0.251	0.300	0.306	0.306	0.307
μ_x (a.u.)	2.920	2.951	2.953	2.953	2.959
μ_y (a.u)	-0.003	0.000	-0.002	-0.001	0.001
μ_z (a.u)	0.089	-0.133	-0.141	-0.136	-0.135

As shown in Tab. 2, the 2.1-0.1 mesh, made up of 624 compares very well with the most refined 1.1-0.1 mesh made of 1712 elements. In particular the E_2 is only 1.44 meV larger than the corresponding value of the finer mesh. Also the molecule dipole moment d_z^2 is only 0.001 a.u. off from the corresponding value, and the NP induced dipole moment μ_x and μ_z differ by 0.006 a.u. with respect to the 1712 tesserae mesh. The μ_x value is, moreover, in very good agreement with the analytic predictions, 2.96 a.u., of the classical image dipole

induced on a metallic sphere by a point charge dipole of magnitude equal to LiCN ground state one and in the same geometry as the LiCN/NP case.

3 LiCN, high fields and Rabi oscillations

To stress test the numerics we applied unphysically high external fields field intensities ranging from 0.005 a.u. ($4.7 \cdot 10^{13}$ W/cm^2) to 0.5 a.u ($4.7 \cdot 10^{17}$ W/cm^2). In this highly non linear regime Rabi-like oscillations between ground and excited states populations appeared, their frequency strongly dependent on the molecule-NP distance. Indeed TDCI[2,3] and real time (RT)-TDDFT[1] were employed to study Rabi oscillations of the resonant state population linked to the dipole switch. In the latter work, RT-TDDFT calculations were not able to reproduce the Rabi oscillations. This failure, a fundamental issue of the conventional Kohn-Sham formulation, has been ascribed to the dynamical detuning induced by the adiabatic approximation to the xc functionals[4] and, recently, to the presence of unphysical multielectron excitations which could be removed by allowing the variation of the number of Kohn-Sham orbitals and of their occupations.[5] We thus studied how Rabi oscillations are described in our formalism and what is the impact on them of a plasmonic NP. Before going into the details of the calculation, however, it is important to stress that in this case we should not look at the results as the predicted behaviour of a real LiCN molecule (for instance we are not including the possibility for the molecule to ionize), nor as the quantum behavior of the NP, but more as the behaviour of a model system made of a given number of energy levels differently coupled to the external field, to the NP, and among themselves.

3.1 LiCN high fields dynamics in vacuum

We first look at the electron dynamics of the LiCN molecule in vacuum. The molecule is oriented along the x direction, the external sinusoidal field polarized along the z direction in resonance with the doubly degenerate charge-transfer second excited state (see S.I. for

GW-BSE excitation levels and absorption spectrum). Except for analysis purposes, the first 15 excited states were included in the dynamics.

In Fig. 3 the time evolution of the applied external field (top panels), of the molecule dipole moment (middle panels) and of the ground and resonant excited states (bottom panels) are reported for 0.005 a.u. (left) and 0.01 a.u. (right) field intensities. The black arrow shows τ_0 which we define as the first time in which the ground state population is 0 for ideal Rabi oscillations of a two level system whose transition dipole is the same as that of LiCN resonant excited state. Namely $\tau_0 = \pi/\omega_R$, where $\omega_R = \vec{E}_0 \cdot \vec{\mu}$. ω_R is the Rabi frequency, E_0 the amplitude of the applied field, and $\vec{\mu}$ is the transition dipole moment of the resonant state. At 0.005 a.u. the system dynamic is almost ideally Rabi-like, with full dipole switching from negative to positive values and population and depopulation of the s resonant excited state. When doubling the field intensity to 0.01 a.u., as shown in the right panel of Fig. 3, the behaviour becomes less ideal, and states different from the ground and excited state ones come into play gaining a finite population.

The role of excited states different from the resonant one becomes more important as the field amplitude further increases as shown in the left panel of Fig. 3. Indeed, when we limit the number of states included in the dynamics to four (ground and excited states up to the resonant one), the ideal dynamics is recovered (furthest right panel in Fig. 3).

3.2 LiCN/NP coupled dynamics: field enhancement and Rabi oscillations

We propagate Eq. 3 and Eq. 13 for the LiCN molecule in proximity of the spherical NP, but at first we take into account only the first four energy levels to obtain a simplified population dynamics (as the results in vacuum suggest) that allows to track the field enhancement of the NP at the different distances by looking at the Rabi frequencies. The effect of including more states in the dynamics is analysed at the end of the section. The external field is polarized along the z-direction, its amplitude is 0.005 a.u. ($4.7*10^{13}$ W/cm^2), and its frequency is

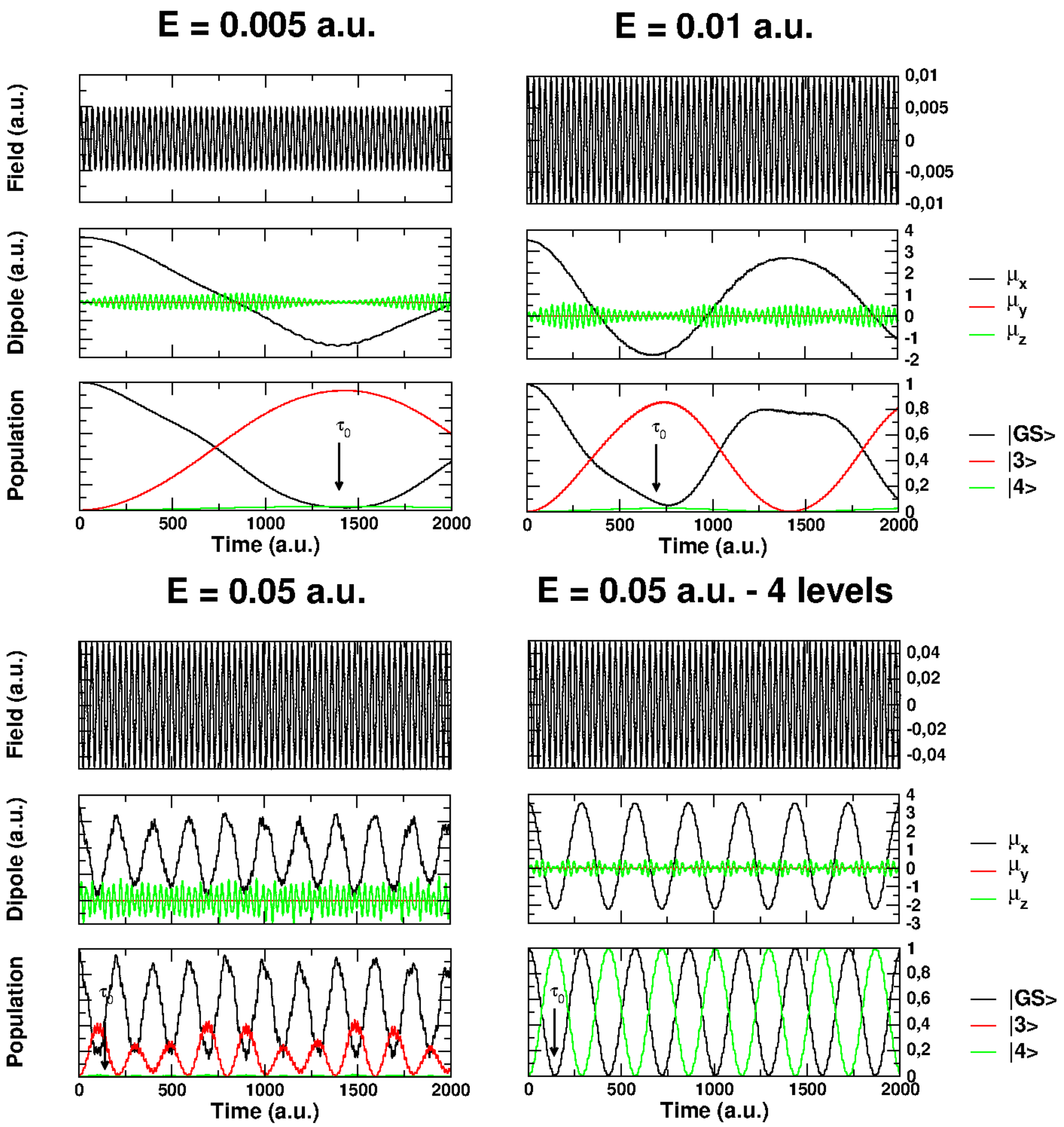

Figure S3: Top panels: LiCN time evolution of the external electric field (upper panels), of the molecule dipole moment (middle panel), and of the populations of ground and resonant excited states for 0.005 a.u. (left) and 0.01 a.u. (right) field intensities. All results refer to calculations involving the first 15 excited states. Bottom panels: time evolution of the external electric field (upper panels), of the molecule dipole moment (middle panel), and of the populations of ground and resonant excited states for 0.05 a.u. field amplitude. The left panels involve the first 15 excited states, while right panel only the first three excited states.

chosen in order to match the modified excitation energies of the dipole-switch excited states which is provided by the preliminary self-consistent equilibration procedure described in the previous subsection.

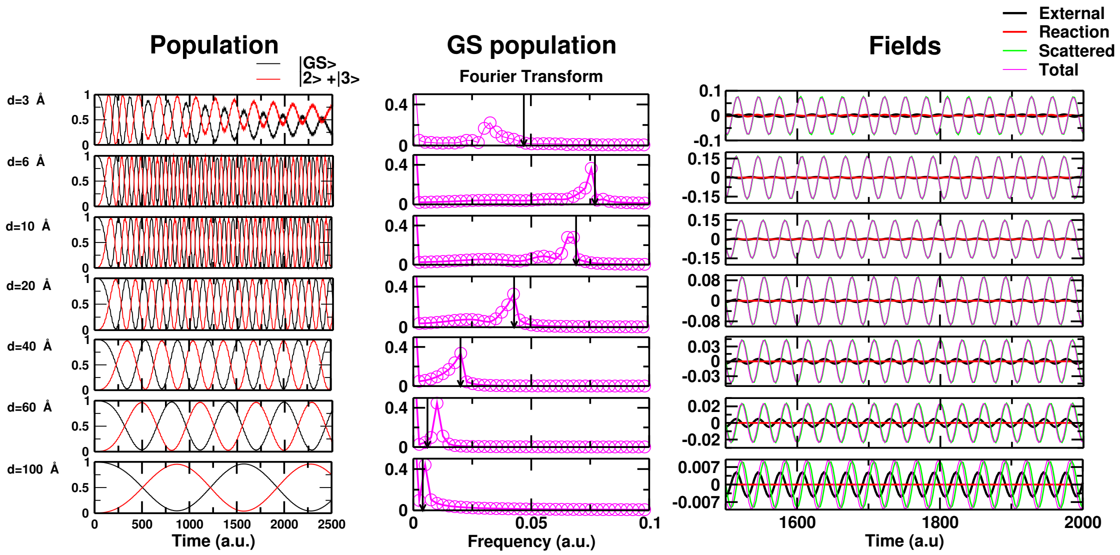

Figure S4: Left panel: population of the ground state (black) and of the excited states in resonance with the external pulse (summed up in red). Central panel: Fourier Transform of the ground state population. The black arrow point at the ideal Rabi frequency expected from the amplitude of the total field at the molecule center. Right panel: z-component of the incident (black), image (red), scattered (green) and total electric fields at the molecule center, for their definitions see text. Going from upper to lower panels the distance between NP and molecule increases from 3Å to 100Å. All quantities are expressed in a.u..

In the left panel of Fig. 4 we report the time evolution of the ground state (in black) and resonant state (in red) populations, in the middle panel the Fourier transform (FT) of the ground state population dynamics, from top to bottom the results for the different molecule/NP distances are reported. The left panel of Fig. 4 shows that, except for the 3 Å case, regular Rabi oscillations are present in the population dynamics. The period of such oscillations increases with the molecule/NP distance which is also detectable from the shift toward lower frequencies of the peak in the FT (middle panel) and is a consequence of the trend of the enhancement of the external field. Indeed the frequency of Rabi oscillations is proportional to the field amplitude along the direction of the resonant state transition dipole moment, the black arrows in the middle panel of Fig. 4, that point at the ideal Rabi

frequencies expected from the value of total field amplitude, are in good agreement with the position of the peaks of the population dynamics Fourier transform. The value of the z-component of the electric field experienced by the molecule is shown in the right panel of Fig. 4. Following the common notation,[6] we split the total field (in magenta) in three contributions: incident (in black), image (red) and scattered (green) field. Scattered fields are those fields produced by the polarization that is induced within the NP by the external field, whereas image fields are due to the polarization that the NP develops responding to the fluctuation of the molecule charge density. The total field amplitude decreases by two order of magnitude increasing the distance from 3 Å to 100 Å, scattered fields give the strongest contribution in all cases. It is worth noting that for the 3 Å distance the image field is comparable to the incident field but still much smaller than the scattered field.

From Rabi frequencies, obtained by Fourier transforming the population dynamics, the field amplification for each distance is computed by inverting the relation $\omega_R = \vec{E} \cdot \vec{\mu}$. At the frequency ω_R of the external pulse, an analytic estimation of the field amplification can moreover be obtained considering a classical dielectric sphere in an uniform external field.[7] The results are listed in Tab. 3, compared with the field amplification obtained from the values of the amplitude of the total field at the molecule center.

Table S3: Amplification of the electric field experienced by the molecule compared to the value extracted by Rabi frequencies and to the analytic model of a dielectric sphere placed in a uniform external field. The dielectric constant of the sphere is given by the value of the Drude-Lorentz dielectric function at the equilibrated molecule resonance energy, specific for each distance. In the $\epsilon = \epsilon(\omega_{d=3})$ case the sphere dielectric constant is kept fixed at the 3 Å distance evalue for all distances.

Dist (Å)	3	6	10	20	40	60	100
Numeric	21.3	34.8	30.7	19.4	8.9	4.8	2.0
From Rabi	14.4	32.7	31.1	18.5	7.6	3.2	2.3
Analytic	13.5	31.7	29.0	18.6	8.7	4.7	1.9
$\epsilon = \epsilon(\omega_{d=3})$	13.5	11.3	9.0	5.3	2.0	0.8	0.5

The analytic model predicts a non monotonic behaviour of the amplification with respect to distance which is also found numerically. This unexpected trend is due to the variation of

the excitation energy for the different NP/molecule distances, which then 'picks' a different value of the NP dielectric function. Indeed, as shown in the last line of Tab. 3, by fixing the value of the dielectric function within the analytic model, the expected monotonic decreasing behaviour is recovered. When comparing the numeric and analytic sets of results it is clearly seen that the model predicts systematically lower values, especially at small distances. The fact that the analytic model can account only for the scattered field contributions could possibly explain this underestimation. The amplification values extracted from Rabi oscillation frequencies are in good agreement with both but are slightly more scattered, most probably this is due to the low resolution of the FT and a better agreement could be reached by longer simulation times. At 3 Å distance the amplification extracted from the Rabi frequency is in much better agreement with the analytic model with respect to the numeric value which may be extremely sensitive to the choice of the point where the field is evaluated for this very small NP/molecule distance.

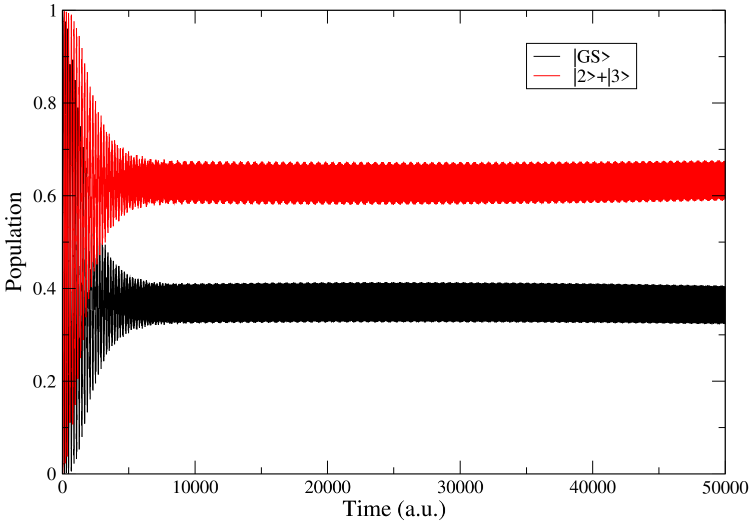

Figure S5: Population of the ground state (black) and of the excited states in resonance with the external pulse (summed up in red) for the 3Å NP/molecule distance.

The 3 Å case is qualitatively different from the rest. As seen from the left panel of Fig. 4 and, for a longer simulation, in Fig. 5, population still oscillates between ground and resonant excited states but, after a transient of about 500 a.u., the oscillations amplitudes are damped around asymptotic population values. The stronger coupling with the NP can explain the

different behaviour of this case. Damped Rabi oscillations around asymptotic populations were also found, for example, in biased semiconductors superlattices[8] and cold trapped ions[9] when the two level system was coupled to other states or to the continuum: in particular the different magnitude of the coupling (with the continuum for the former and with an auxiliary state for the latter) explained the asymmetry in the asymptotic populations of the ground and resonant states. Also in our case the NP/molecule proximity may enhance the differences in the NP/molecule coupling for the two states leading to the different asymptotic populations.

The inclusion of excitations up to 10 eV (20 states) has strong effects on the population dynamics, as shown in Fig. 6. For the 6 Å and 10 Å distances, when the field experienced by the molecule is maximally enhanced, the population of the resonant excited states is almost entirely quenched and the population is transferred to higher energy excitations. Slowly, as the distance increases and the field decreases, a two-states Rabi-like oscillatory behaviour is recovered.

4 LiCN active space size convergence

The convergence of the population dynamics with respect to the active space size, i.e. the number of excited states included in the dynamics, depends on the system under investigation, due to system-dependent spacing of the excitation energies and strengths of the transition dipoles, on the NP-molecule distance and on the magnitude of the external field. In Fig. 7 we report the population dynamics of the dipole switch states for different number of excited states included in the dynamics, in both high and standard field regimes, for selected NP-molecule distances. For low fields intensities (right panel) the dynamics is not strongly sensitive to the number of states included in the dynamics even for 3 Å NP-molecule distance, where the field amplification is very large. On the opposite, as mentioned in the previous sections, when the field intensity is very high, care must be put in converging the

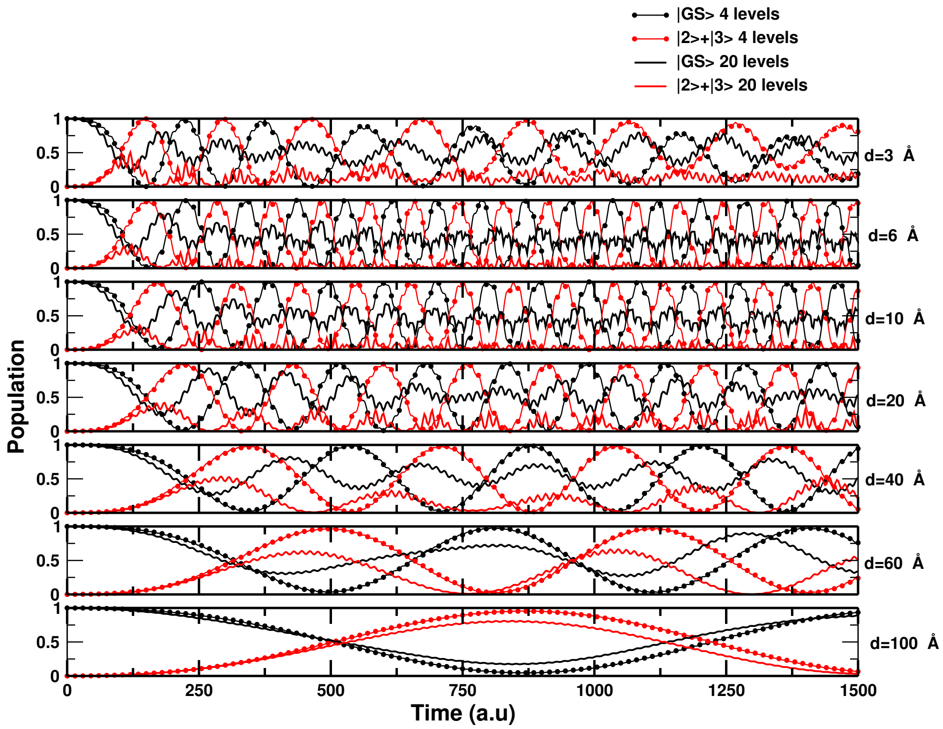

Figure S6: LiCN: time evolution of the population of ground state and of the excited states in resonance with the external pulse. Dotted black and red lines: ground state and excited state respectively for the four levels dynamics. Full black and red lines: ground state and excited state respectively for the twenty levels dynamics.

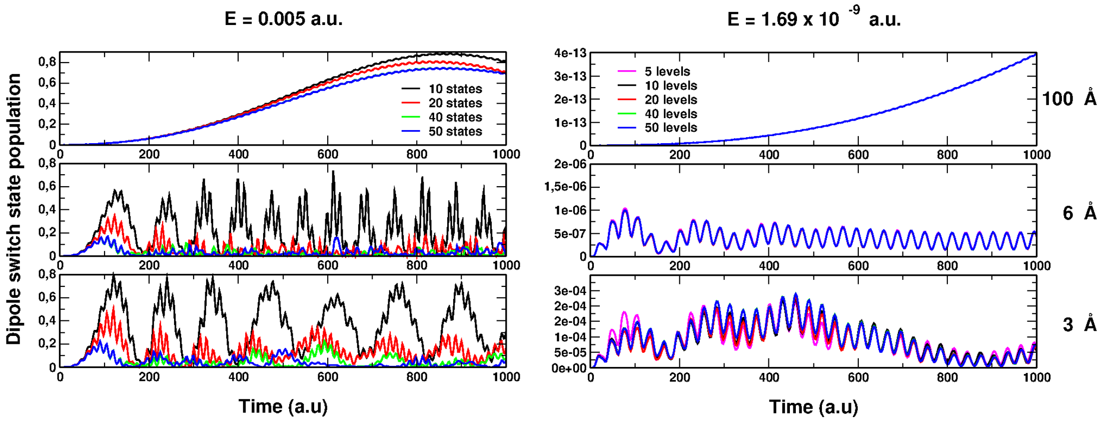

Figure S7: LiCN: population dynamics of the dipole switch states including a growing number of excitations in the dynamics. Left panel: high field regime. Right panel: standard field regime. Top panels: 100 Å NP-molecule distance; middle panels: 6 Å NP-molecule distance; bottom panels: 3 Å NP-molecule distance.

size of the active space taking into account the NP-molecule distance. Indeed whereas for 100 Å distance a good convergence is found also including only the first 10 excitations, for shorter distances 40 excitations seem enough only for the first 200 a.u.. As already discussed, at such high field intensities, other effects, such as molecule ionization and tunneling, should be included for a realistic description of the elctronic dynamics. Indeed, such high fields regimes are not of interest for the applications that we envisage, nevertheless calculations in this regime could still be useful for assessing the effective field amplification (over the entire molecule volume) through the analysis of the Rabi oscillation frequency.

5 PNA GW-BSE electronic structure and optical properties

PNA G_nW_n electronic gap is 8.7 eV, its ionization potential (IP) being 8.5 eV and its electron affinity (EA) -0.3 eV. For comparison, at the DFT level, the electronic gap is 4.8 eV, the IP is 6.7 eV and the EA is 1.9 eV. Experimentally, the IP ranges between 8.34 eV and 8.85 eV, and the EA ranges between 0.75 eV and 0.95 eV.[10]

Experimental visible-UV absorption spectra[11] report a first excitation with finite oscillator strength at 4.24 eV in rough agreement with our first excitation at 4.77 eV.

6 PNA Determination of an optimal tesselation

An optimal tessellation of the NP surface is critical for an accurate and computationally affordable calculation. We generated non uniform tesselations with decreasing tesserae dimension when approaching the cone tip using the open source 3D finite element mesh generator Gmsh[12] by fixing the desired mesh element size (mes) at specific points on the surface. In Fig. 8 the points were the mes has been fixed are shown. As the cone is obtained by a 360 degree rotation of half of its section, all the equivalent points in terms of such rotation have

the same mes. In particular we fixed the mes of the top surface points (D and equivalent

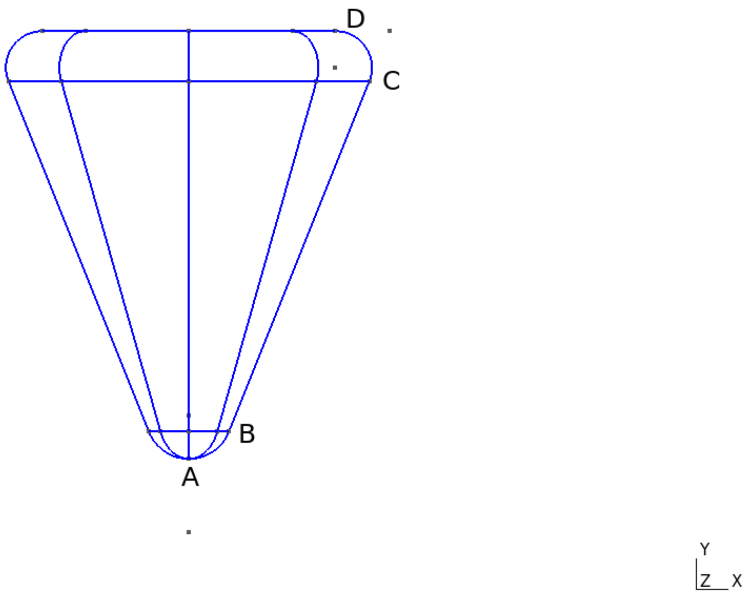

Figure S8: Section of the tip NP, with the points were the mes has been fixed.

points) at 1.06 nm, of the edge of the top rounding (C and equivalent) at 0.85 nm, and varied those of the edge of the bottom rounding (B and equivalent) and of the tip (point A). First, keeping fixed the mes at point B at three values (0.11 nm, 0.08 nm and 0.05 nm), the mes at A was varied between 0.02nm and 0.01 nm. The static effect on the excitation energy, reported in Tab. 4, is very small.

For the different combinations of MES the coupled equation of motion of the NP+molecule system was propagated at the A NP-molecule geometry, namely with the tip pointing to the center of the benzene ring. In the propagation the first 4 excited states were taken into account, the external field, of 0.005 a.u., being in resonance with the first bright excitation. The results for the population of the resonant excited state are shown in Fig 9. As can be seen by comparing couples of curves at fixed mes(B), at 0.02 nm the mesh element size at point A seems converged.

Fixing the MES of the A point to the converged value 0.02 nm, the MES at B is sys-

Table S4: Tested mesh element sizes (mes) for B (and equivalent points), and for the tip point A, see also Fig. 8. Corresponding total tesserae number N_t and renormalized excitation energy of the first bright excited state E_3.

mes(B) (nm)	mes(A) (nm)	N_t	E_3 (eV)
0.11	0.02	1136	4.748421
0.11	0.01	1568	4.748397
0.08	0.02	1628	4.748153
0.08	0.01	2040	4.748128
0.05	0.02	2428	4.748046
0.05	0.01	3062	4.748026
0.04	0.02	3626	4.747793
0.03	0.02	4898	4.747982

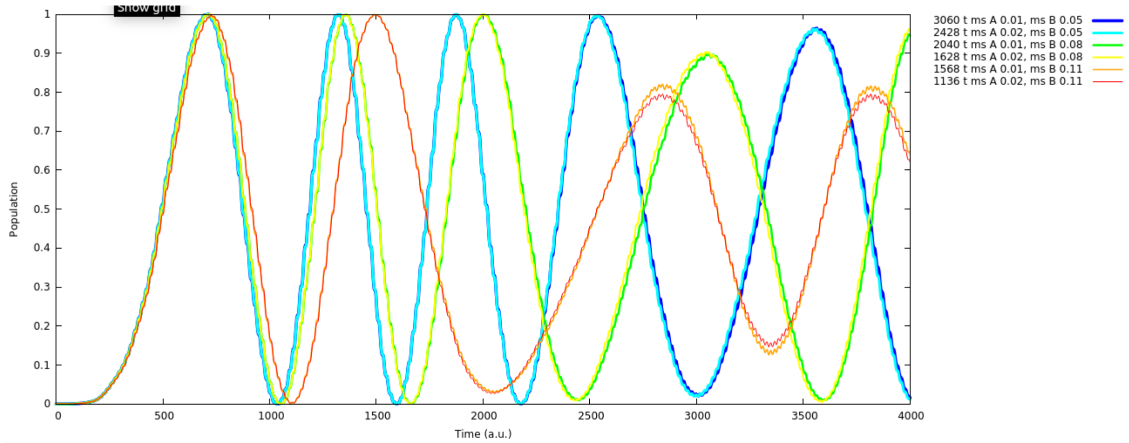

Figure S9: Convergence of the excited state population dynamics with respect to the mes at point A.

tematically decreased down to 0.03 nm. The resonant state population dynamics for the different meshes, shown in Fig. 10, show that convergence is reached for a B MES of 0.05 nm.

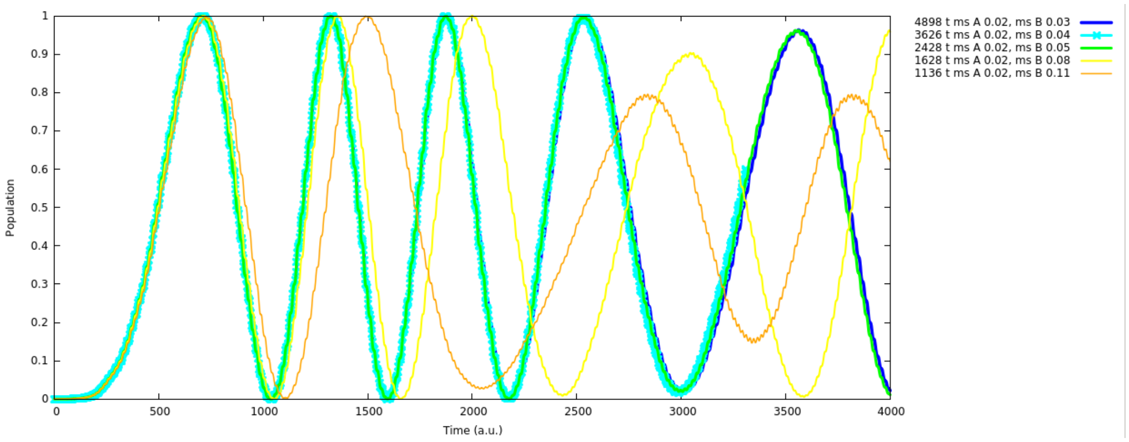

Figure S10: Convergence of the excited state population dynamics with respect to the mes at point B.

7 PNA 18 vs 4 levels dynamics

In the case of LiCN a simplification of the population dynamics and a Rabi-like behaviour (with the exception of the 3 Ådistance case) could be obtained once the number of the excitations involved in the dynamics was reduced. A different behaviour is found for the PNA case where including excited states up to 8 eV (first 17 excitations) does not lead to a much different dynamics with respect to including only the first 3 excitations as shown in Fig. 11 for the case of geometry A.

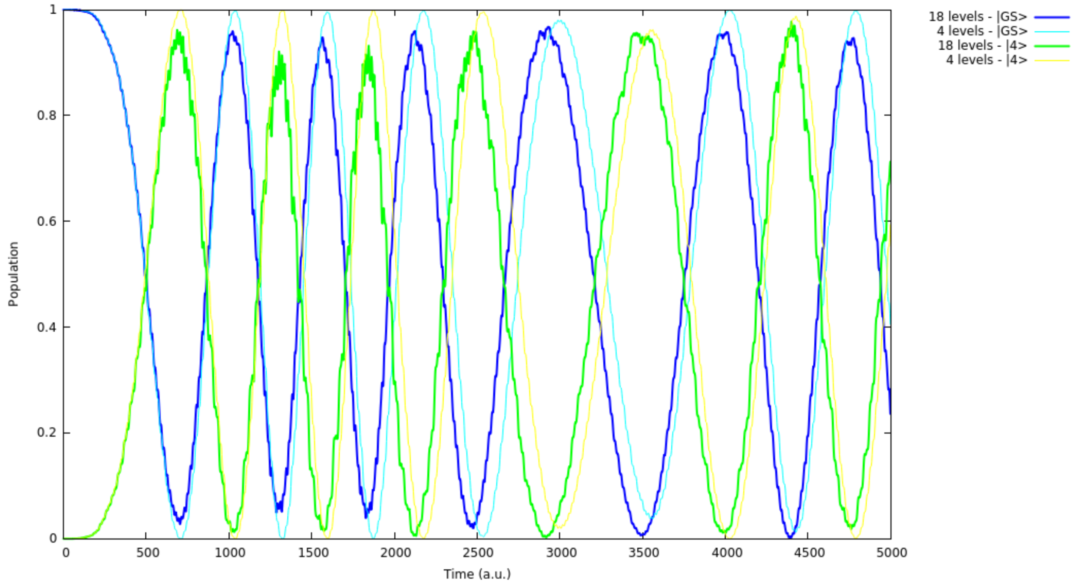

Figure S11: Ground state and excited state population dynamics for calculations involving 18 and 4 levels. The PNA molecule is located at geometry A with respect to the NP.

References

(1) Raghunathan, S.; Nest, M. Critical examination of explicitly time-dependent density functional theory for coherent control of dipole switching. *J. Chem. Theory Comput.* **2011**, *7*, 2492–2497.

(2) Krause, P.; Klamroth, T.; Saalfrank, P. Time-dependent configuration-interaction calculations of laser-pulse-driven many-electron dynamics: Controlled dipole switching in lithium cyanide. *J. Chem. Phys.* **2005**, *123*, 074105.

(3) Tremblay, J. C.; Klamroth, T.; Saalfrank, P. Time-dependent configuration-interaction calculations of laser-driven dynamics in presence of dissipation. *J. Chem. Phys.* **2008**, *129*, 084302.

(4) Fuks, J. I.; Helbig, N.; Tokatly, I. V.; Rubio, A. Nonlinear phenomena in time-dependent density-functional theory: What Rabi oscillations can teach us. *Phys. Rev. B* **2011**, *84*, 075107.

(5) Zang, X.; Schwingenschlögl, U.; Lusk, M. T. Identification and Resolution of Unphysical Multielectron Excitations in the Real-Time Time-Dependent Kohn-Sham Formulation. *Phys. Rev. Lett.* **2020**, *124*, 026402.

(6) Aravind, P. K.; Metiu, H. Use of a perfectly conducting sphere to excite the plasmon of a flat surface. 1. Calculation of the local field with applications to surface-enhanced spectroscopy. *The Journal of Physical Chemistry* **1982**, *86*, 5076–5084.

(7) Jackson, J. D. *Classical Electrodynamics*; John Wiley & Sons Inc: Hoboken, New Jersey, U.S., 1998.

(8) Abumov, P.; Sprung, D. W. L. Interminiband Rabi oscillations in biased semiconductor superlattices. *Phys. Rev. B* **2007**, *75*, 165421.

(9) Di Fidio, C.; Vogel, W. Damped Rabi oscillations of a cold trapped ion. *Phys. Rev. A* **2000**, *62*, 031802.

(10) Jackson, J. D. *NIST Chemistry WebBook, NIST Standard Reference Database Number 69*; P.J. Linstrom and W.G. Mallard, Eds, 2021.

(11) Millefiori, S.; Favini, G.; Millefiori, A.; Grasso, D. Electronic spectra and structure of nitroanilines. *Spectrochim. Acta, Part A* **1977**, *33*, 21–27.

(12) Geuzaine, C.; Remacle, J.-F. Gmsh: a three-dimensional finite element mesh generator with built-in pre- and post-processing facilities. *Int. J. Numer. Meth. Engng.* **2009**, *79*, 1309–1331.
